# The Foreign Journals: Midwifery, and Diseases of Women and Children

**Published:** 1842-07

**Authors:** 


### MIDWIFERY AND DISEASES OF WOMEN AND CHILDREN.

*Occurrence of confluent Smallpox in a Child before birth, without any similar eruption appearing on the Mother, who had been vaccinated.* By Dr. C GNOLI.

Rosa Galvani, 37 years old, a healthy woman who had been vaccinated successfully when an infant, was delivered of a male child on June 3, 1841. The labour was easy and the mother recovered without any bad symptoms, but the infant was an eight months' child; labour seemed to have been induced by the smallpox with the pustules of which the child was covered. At birth the child was in a comatose state, from which it was relieved by allowing some blood to flow from the umbilical cord. On the second day the pustules appeared at their height, on the fifth day maturation began, but on the sixth a black spot showed itself in the centre of each pustule and the child was attacked with febrile symptoms, subsultus, and trismus. On the seventh and eighth days this condition became aggravated, and the child died at 3 a. m. of the ninth day after birth.

When interrogated about her own health, the mother stated that about a week before delivery she felt generally ill, was feverish, lost her appetite, and suffered much from heat in the stomach, but not so severe as to make her seek for medical advice.

*Bulletino delle Scienze Mèdiche di Bologna. Aug. and Sep.* 1841.

---

*Experiments on the best Agent to be employed in Cauterizing Ulcerations of the Cervix Uteri.* By M. LISFRANC.

Two agents have hitherto been employed indifferently for this purpose, the solid nitrate of silver, and a brush dipped in the acid proto-nitrate of mercury, both which remedies have brought about the cicatrization of the ulcers. M. Lisfranc has recently instituted experiments with a view to ascertain the comparative merits of the two applications. From these experiments it results that cauterization with the acid nitrate of mercury has seldom excited any discharge of blood, while blood frequently flowed in greater or less quantity after the employment of nitrate of silver. From this fact the inference would be that whenever the ulceration is accompanied (as is frequently the case) with a certain degree of engorgement of the body of the uterus the nitrate of silver must not be used.

This statement of the comparative effects of the two caustics is supported by the results of seventy-two cauterizations of the çervix uteri, forty-four of which were made with the nitrate of silver, in thirty-one of which there was a discharge of blood afterwards, while that occurrence took place in three only out of twenty-eight instances in which the acid nitrate of mercury was employed.

*Bulletin Générale de Thérapeutique. Feb.* 28, 1842.

---

*Indentation of the Os Frontis of a new-born Infant, produced by an exostosis between the fourth and fifth lumbar vertebra of the Mother.* By Dr. Duntzer, of Cologne.

A rachitic woman, only four feet three inches high, who had passed through three difficult labours, and the conjugate diameter of whose pelvis measured only three and a quarter inches, suffered during her fourth pregnancy from dyspepsia and derangement of the hepatic functions. These symptoms were relieved, and the general health of the patient became very good, but in the last two months of pregnancy she complained of a dull pain and sense of pressure in the situation of the last lumbar vertebra. The discharge of the urine and fæces was not impeded, but the patient was prevented by the pain from lying on her back. When labour came on, the passage of the head was attended with extreme difficulty, and could be effected only by the application of the long forceps. As soon as the child was born a large depression of the frontal bone was seen, of an oval form, extending transversely from the superciliary ridge to the coronal suture, being two inches and a half long, one inch and a half broad, and one inch in depth, but not accompanied with either fissure or fracture of the bone, or with sugillation or any unusual redness of the skin. The child's health continued good, though it was in a state of asphyxia at birth. In the course of a few days the depression of the bone was lessened, in three months' time it had disappeared, and at the time of this report, the child being then six months old, the curve of the os frontis was nearly the same on both sides. On examining the mother per vaginam, an oval exostosis of the size of a pigeon's egg was detected between the fourth and fifth lumbar vertebra, and the promontory of the sacrum projected somewhat more than natural. To the case are appended references to others in which similar injuries occurred to the head of the child, and which are of considerable importance in a medico-legal point of view.

*Neue Zeitschrift für Geburtskunde.* 11 Bd. 3 Heft, S. 360.

---

*Case in which the Placenta was retained for eleven weeks after the birth of a premature Child.* By Dr. Schöller.

A poor woman, 37 years old, having overexerted herself, was taken in labour in the fifth month of her second pregnancy. A midwife who was summoned tore the funis in her endeavour to remove the placenta, and an accoucheur who was then sent for could not succeed in extracting it. The woman now resumed her usual occupations till she was compelled to seek medical advice by the occurrence of hemorrhage from the uterus.

Ten weeks after her miscarriage she applied to Dr. Schöller, who on making a vaginal examination found the cervix uteri thick, the os sufficiently open to admit the finger, and the uterus itself felt large and as though it contained a foreign body. The woman was ordered to remain in bed, and to take gr. x. of ergot of rye every two hours. After the administration of twelve doses pains like those of labour came on, and were followed by the expulsion of coagula mixed with fibrous and membranous matters, and having a very offensive odour.

Dr. Schöller now fancied that the case was at an end, and supposed that this was an instance of real absorption of the placenta, but after the lapse of some days, having administered a purgative, pains came on in the abdomen and recurred periodically for some hours until a thick mass was expelled from the uterus. This mass was ascertained to be the placenta, which had not undergone the slightest decomposition, was hard, surrounded by a coating of fibrine, and shrunk to the size of half a goose's egg. On a section it presented the peculiar structure of the placenta. The patient did well.

[The case is interesting in connexion with the question which has been much debated of the occurrence or non-occurrence of absorption of the placenta in cases where it is retained for a considerable time in the uterus.]

*Medicinische Zeitung.* *Feb.* 16, 1842.

---

*Cure of Acute Hydrocephalus by the spontaneous Discharge of Water from the Ear.* By Dr. RIECKE.

A boy, two years and a half old, well nourished, robust, and with a prominent forehead, became unwell on the 8th December, 1841. The symptoms were those of an inflammatory cerebral affection, attended with fever. The disease had made considerable progress by the 14th, which was the day on which the author first saw the patient. The prognosis was the more unfavorable, inasmuch as the great fontanelle was unclosed to the extent of an inch in diameter. Every means was put in requisition—bloodletting, calomel internally, mercurial friction on the nape of the neck, blisters, cold applications to the head—without benefit. The disease went on increasing, and seemed on the 26th to have reached its height. The little patient lay powerless and stupid, the head and face flushed and hot, there was grinding of the teeth, the pupils were relaxed and insensible to light. The child had ceased to scream. The diuretic medicines resorted to latterly had failed to promote the urinary secretion. While in this state, on the 20th day of the disease, there flowed from the right ear such a quantity of pure and limpid fluid as drenched thoroughly the child's neckerchiefs. On the same evening, the patient was much relieved. By the use of diuretics, the flow of urine was now maintained, in a copious current, during many days. The coma, in which the child had been for some time, disappeared; the pupil regained motion; in six weeks the little patient was completely cured.

Another case of acute hydrocephalus is given, in which a similar discharge, but to a less extent, took place. This was on the 20th of February, 1834, and the child recovered from that attack ; but subsequently on the 6th of December, 1836, had a second attack of the same disease, from which it also recovered, though on the last occasion no watery discharge from the ear took place.

*Casper's Wochenschrift.* No. xviii. *April* 30, 1842.

